# Magnetically Induced Alignment of Natural Products for Stereochemical Structure Determination via NMR

**DOI:** 10.1002/anie.202004881

**Published:** 2020-06-03

**Authors:** Niels Karschin, Klaus Wolkenstein, Christian Griesinger

**Affiliations:** ^1^ Department for NMR-based Structural Biology Max-Planck Institute for Biophysical Chemistry Am Fassberg 11 37077 Göttingen Germany; ^2^ Department of Geobiology, Geoscience Centre University of Göttingen Goldschmidtstraße 3 37077 Göttingen Germany

**Keywords:** anisotropic NMR parameters, configuration determination, density functional calculations, magnetically induced alignment, NMR spectroscopy

## Abstract

Anisotropic NMR has gained increasing popularity to determine the structure and specifically the configuration of small, flexible, non‐crystallizable molecules. However, it suffers from the necessity to dissolve the analyte in special media such as liquid crystals or polymer gels. Generally, small degrees of alignment are also caused by an anisotropic magnetic susceptibility of the molecule, for example, induced by aromatic moieties. For this mechanism, the alignment can be predicted via density functional theory. Here we show that both residual dipolar couplings and residual chemical shift anisotropies can be acquired from natural products without special sample preparation using magnetically induced alignment. On the two examples of the novel natural product gymnochrome G and the alkaloid strychnine, these data, together with the predicted alignment, yield the correct configuration with high certainty.

## Graphical abstract

**Properly aligned**: Residual dipolar couplings and residual chemical shift anisotropies can be acquired from natural products without special sample preparation using magnetically induced alignment. On the basis of two examples—the novel natural product gymnochrome G and the alkaloid strychnine—these data, together with the predicted alignment, yield the correct configuration with high certainty.

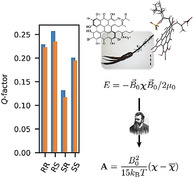

In the field of natural products, the determination of molecular structure is one of the fundamental steps in characterizing the isolated compounds. Unfortunately, due to the structural complexity and conformational heterogeneity of many natural products, they cannot be crystallized, calling for NMR. Yet, the unambiguous interpretation of experimental NMR data can be difficult and structural misassignments happen regularly.^1^ To increase the robustness of structure determination, we believe it is advisable to use different approaches to come to the same conclusion whenever it is possible. Also, the combination of spectroscopic data with molecular modeling has proven to be a valuable tool in the elucidation of the correct structure, and many techniques rely on results from density functional theory (DFT) and molecular mechanics (MM) simulations.^2^ One example of such an approach is the use of anisotropic NMR parameters. These parameters appear when the orientational distribution of the analyte molecule with respect to the magnetic field is non‐uniform, a condition which is termed alignment. Since the chemical shift is anisotropic, the motionally averaged shift measured in the spectrum is different under aligned conditions, and this difference is called residual chemical shift anisotropy (RCSA). Similarly, dipolar couplings, which average to zero under isotropic conditions, make a contribution to the overall coupling in aligned samples, and the measured coupling is changed by this residual dipolar coupling (RDC). These parameters then provide orientational constraints with respect to a molecular frame. Structural models for all candidate isomers are generated via MM/DFT and the agreement of the data with these models is evaluated. This often allows discriminating between different possible diastereomers. In the established protocols, alignment is achieved by introducing the analyte into an anisotropic medium, such as liquid crystals, filamentous phages, or deformed polymer gels.^3^ However, this process comes with certain drawbacks, especially if only small amounts of analyte are available (as it is common for natural products). First, any transfer of the analyte suffers from inevitable sample losses; second, recovery of the analyte from anisotropic media can be difficult; third, it prohibits the acquisition of any new experiments under normal, isotropic conditions for the time being; and fourth, the alignment tensor needs to be fitted and can yet not be sufficiently accurately predicted.

Therefore, we set out to obtain alignment without an alignment medium. A simpler way to achieve anisotropic conditions is by exploiting the alignment that is induced by the external magnetic field. This so‐called self‐alignment occurs if the analyte molecule has an anisotropic magnetic susceptibility. It scales with the square of the external magnetic field *B*_0_.^4^ It is most commonly used in the investigation of metal‐binding proteins, where paramagnetic metal centers (for example, transition metals or lanthanides) with substantially anisotropic susceptibilities lead to large degrees of alignment, and the isotropic reference experiment can be acquired on diamagnetic counterparts (for example, Zn, La, or Lu).^5^ For diamagnetic cases, aromatic systems are the most relevant sources for anisotropic susceptibilities. RDCs and RCSAs can be determined by performing field‐dependent measurements, but the effects are typically much smaller than for paramagnetic metal centers. RDCs have already been determined this way, both for biomacromolecules such as proteins and oligonucleotides as well as for small molecules with large aromatic systems such as porphyrins.5e, 6 However, to our knowledge, the measurement of RCSAs and the use of either RDCs or RCSAs for small molecule structure (conformation and configuration) determination through self‐alignment have not been reported so far. In this work, we show the feasibility of this approach on two molecular examples. First, gymnochrome G (**1**; Scheme 1) is a previously unknown marine natural product isolated from the deep‐sea crinoid *Hypalocrinus naresianus*, featuring a large, proton‐deficient aromatic system and two side chains with one stereocenter each. Its aromatic system with eight annealed rings leads to an unusually large degree of alignment and makes this an ideal target for this approach. While other compounds from this organism have been elucidated,^7^ **1** has not been described in the literature so far. We corroborate the configuration using established NMR‐based techniques2b, 8 as (2′*S*,2′′*R*) (see the Supporting Information, Section S1). Second, to show that this method is more universally applicable, we apply it to strychnine (**2**), a well‐studied alkaloid with only a single aromatic ring. Despite its relatively low degree of self‐alignment, we were able to identify the correct configuration from the set of its diastereomers with high confidence.

**Scheme 1:**
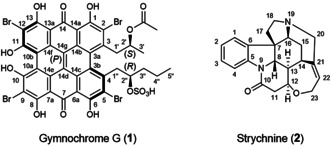
Constitution, configuration, and numbering of the investigated structures.

We measured simple ^13^C and ^1^H 1D‐NMR spectra of both compounds at different magnetic fields (between 400 and 950 MHz proton frequency) and extracted ^13^C chemical shifts and ^1^H–^1^H couplings. In the case of **2**, we also acquired CLIP‐HSQC spectra^9^ to determine ^13^C–^1^H and ^1^H–^1^H couplings. Both the chemical shift (in ppm) and the coupling (in Hz) are composed of two components: the isotropic component, which is constant with the magnetic field, and the anisotropic component, which has a square dependence on the magnetic field. This desired anisotropic component, that is, the RCSA or RDC, can be determined by a simple fitting procedure (for details, see the Supporting Information, Section S2 e). Since these effects are very small (0.1–1 Hz), any source of error has to be carefully excluded. The chemical shift is very sensitive to the experimental conditions, so the perturbation of ^13^C chemical shifts could not be associated to alignment alone. As the spectra at different fields were acquired at different spectrometers on different days, we identified the following systematic errors for ^13^C chemical shifts: first, differences in sample temperature due to imperfections in temperature calibration and different decoupling powers on the order of 0.2 K; second, slow degradation of the sample over the course of time; and third, the referencing. These contributions could be eliminated using basic linear algebra. We postulate that all these errors can be described as linear perturbations as a function of temperature *T*, some degradation coordinate *ξ* (which is not necessarily proportional to time), and a constant *c* quantifying the amount of misreferencing:

(1) $$\vec{\delta }={\vec{\delta }}_{\mathrm{corr}}\left(B_{0}\right)+T{\vec{\delta }}_{T}+{\xi \vec{\delta }}_{\mathrm{degrad}}+{c\vec{\delta }}_{ref},$$

where the five vectors represent the measured chemical shift, the corrected $B_{0}^{2}$ ‐dependent chemical shift, and the chemical shift contributions from temperature, degradation, and misreferencing, respectively. Each assigned ^13^C shift corresponds to one component of these vectors, making them 30‐dimensional for **1** and 21‐dimensional for **2**. For temperature and degradation, we quantified the relative effect (that is, ${\vec{\delta }}_{T}$ and ${\vec{\delta }}_{\mathrm{degrad}}$ ) by acquiring spectra at the same field, but at different temperatures as well as over the course of several weeks; these measurements also confirmed that the assumption of linearity in Equation (1) is a reasonable approximation. Misreferencing is trivial in the sense that every peak is shifted by the same amount and therefore, all elements of ${\vec{\delta }}_{ref}$ are equal. Now, instead of attempting to correct the measured chemical shifts by determining the exact values of *T*, *ξ*, and *c* for each spectrum, another approach proved to be much more effective. We performed an orthonormal transformation of the chemical shift vectors, using the temperature, degradation, and referencing vectors as the first basis vectors. As a consequence, all following base vectors are orthogonal to the perturbation vectors and are therefore not affected by them. Equation (1) then changes as follows:

(2) $$\vec{\delta }={\vec{\delta }}_{\mathrm{corr}}+T\left(\begin{array}{c} \delta_{T,1}\\ 0\\ 0\\ 0\\ \vdots \end{array}\right)+\xi \left(\begin{array}{c} \delta_{\mathrm{degrad},1}\\ \delta_{\mathrm{degrad},2}\\ 0\\ 0\\ \vdots \end{array}\right)+c\left(\begin{array}{c} \delta_{ref,1}\\ \delta_{ref,2}\\ \delta_{ref,3}\\ 0\\ \vdots \end{array}\right)$$

It is easy to see that only the first three components are affected by temperature, degradation, and misreferencing, which have to be discarded. The remaining components are now free from these systematic errors and could be used for structure determination. Only after this procedure, there is reasonable agreement of the chemical shifts with the square‐field dependence of the anisotropic parameters and of the resulting ^13^C RCSAs with the structural models. We attempted to measure ^1^H RCSAs as well, which are expected to be roughly the same size as ^13^C RCSAs (in Hz). However, the proton chemical shift measurement is much less accurate due to broader lines and homonuclear couplings, so acquiring ^1^H RCSAs remained unsuccessful. Couplings are much less sensitive to experimental conditions, so the extraction of the anisotropic component did not require any pre‐processing. The biggest issue are peak distortions. For **1**, the HH‐couplings in the ^1^H 1D‐NMR have to be well resolved and any overlapping peaks have to be discarded. For **2**, peak overlap in the HSQC is less of an issue due to the additional carbon dimension. However, residual long‐range correlations from neighboring protons can lead to significant peak distortions especially in the aromatic region, and generally, the data is more sensitive to errors, as the alignment is smaller. Therefore, the deviation of the data points from the square‐field dependence were used to estimate an error to be used as a weighting factor for both RDCs and RCSAs. This reduces the amount of human bias, as only three aromatic HSQC peaks with obvious distortions had to be discarded.

The degree of alignment is described by a traceless symmetric alignment tensor ***A*** in the molecular frame, which, among others, determines the size of RDCs and RCSAs:^10^

(3) $${\Delta \nu }_{\mathrm{RDC}}=-\frac{3\gamma_{I}\gamma_{S}\mu_{0}\hbar }{8\pi^{2}{∥\vec{R}∥}^{5}}\;\left({\vec{R}}^{T}A\;\vec{R}\right),$$

(4) $${\Delta \delta }_{\mathrm{RCSA}}=\mathrm{tr}\left(A\delta \right),$$

where *γ* are the gyromagnetic ratios of the coupling nuclei, $\vec{R}$ is the internuclear vector between them, and ***δ*** is the chemical shift tensor of the nucleus in question. Generally, anisotropic NMR data do not provide individually interpretable structural constraints, but they are used in their entirety and validated against different structural models. These models are generated for each possible configuration using molecular modeling. In the case of **1**, the aromatic system is inherently chiral, and its configuration was determined via electronic circular dichroism (ECD) to be “propeller”‐(*P*) (see the Supporting Information, Section S1 g). Therefore, due to the two side‐chain stereocenters 2′ and 2′′, there are four possible diastereomers. We conducted a MM‐based conformational search of the side‐chain torsions to generate a conformational ensemble for each diastereomer, containing between 13 and 33 conformers.^11^ For **2**, we assumed no conformational flexibility, and not all combinations of configurations at the six stereocenters are feasible due to the multiple fused and bridged rings. We generated 22 diastereomers and their 3D structure using MM‐based methods, and cross‐checked with published structures of strychnine diastereomers.^12^ All conformations for both **1** and **2** were geometry‐optimized and chemical shift tensors as well as magnetic susceptibility tensors were predicted,^13^ all using the B3LYP functional^14^ and different Jensen basis sets.^15^ For **1**, Boltzmann averaging of the molecular properties was done using thermally corrected free energies. All DFT calculations were performed in Gaussian 09.^16^

We applied two approaches, one in which the DFT‐calculated alignment tensor was used for the validation and another where the alignment tensor was fitted to the conformational ensemble and the experimental values instead. For the latter approach, having multiple conformers as in **1** requires a common frame to be determined first.^17^ In this case, it was natural to use the aromatic system to superimpose the conformers, since it is the source of alignment. With the known experimental data and the calculated structural information from the models (***δ*** and $\vec{R}$ ), the tensor components are determined by finding the least‐squares solution of Equations (3) and (4), and the residuals are used to calculate a *Q*‐factor,^18^ which serves as a measure for the quality of the fit. The configuration which provides the best fit, that is, the lowest *Q*‐factor, is assumed to be the correct solution. By contrast, in the first approach with the DFT‐calculated alignment tensor, the alignment can simply be calculated according to the relation ***A***=(***χ***−*χ*_iso_⋅**1**)*B*_0_^2^/(15*k*_B_ T) using the DFT‐predicted susceptibility tensor.^19^ This enables the complete prediction of anisotropic NMR data based on DFT without the need of any fitting procedures (see the Supporting Information, Section S4 a). Indeed, these predicted data are in excellent agreement with the experimental anisotropic NMR data. A correlation of experimental and predicted anisotropic data for **2** is shown in Figure 1 and can be evaluated quantitatively by a *Q*‐factor. Figure 2 shows the *Q*‐factors for the possible configurations of **1** and **2** for both methods of obtaining the alignment tensor. In all cases, the true solution ((2′*S*,2′′*R*) for **1**, (7*R*,8*S*,12*S*,13*R*,14*R*,16*S*) for **2**) is correctly identified by the lowest *Q*‐factor. Generally, the *Q*‐factor is lower for fitted compared to predicted alignment tensors, as more fitting parameters always lead to smaller residuals. Yet, more important than the absolute values of *Q* are the power of the two approaches to discriminate configurations. In the case of **2**, the use of predicted alignment tensors clearly increases the degree of discrimination, as the *Q*‐factors of the incorrect configurations increase much more than the one of the correct diastereomer: The ratio between the second‐smallest and the smallest *Q*‐factor is only 1.22 for the fitted tensor approach, while it improves to 1.57 by using the predicted tensor. To get a more quantitative measure of the confidence of our result, we performed a bootstrap resampling^20^ to evaluate a confidence level. Here, the increase in confidence of the result for **2** by using predicted alignment becomes obvious, as the confidence increases from around 77 % to 99.2 % (see the Supporting Information, Figure S5). This trend has a simple explanation: when the alignment tensor is fitted, it gives the system some leeway when fitting the data to an incorrect structure, thus reducing the *Q*‐factor and therefore improving the agreement for this incorrect model. For **1** however, the confidence goes from 96 % (fitted) to 88 % (predicted), so there seems to be no advantage in using the predicted tensor. We hypothesize that this is caused by the structural similarity of the possible configurations, which all share the large aromatic system that is the source of alignment. The many data points available in this system with comparatively large anisotropic effects already characterize the alignment tensor very well, while there are relatively few data points and marginal contribution to alignment in the relevant side‐chain regions. The data from the aromatic ring therefore dominates the fitting procedure and the differences between the four diastereomers are small. Also, there is more uncertainty in the structural model of **1** due to the conformational flexibility of the side chains, that is, the regions containing the stereocenters and the conformational averaging using calculated free energies. It is plausible that this uncertainty masks the expected improvement from the predicted alignment tensor compared to the fitted tensor. If the energy is calculated in a different way, for example, without thermal corrections or with a different basis set, the confidences change in the order of 10 %. All analysis and data evaluation were performed using Python3 relying heavily on the SciPy ecosystem.^21^

**Figure 1:**
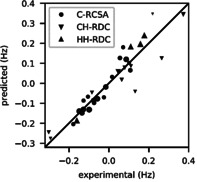
Correlation plot of experimental and DFT‐predicted anisotropic data for the correct diastereomer of **2**. The size of the marker corresponds to the weight of the data point. The values are extrapolated to a field of 23.49 T (1 GHz proton frequency).

**Figure 2:**
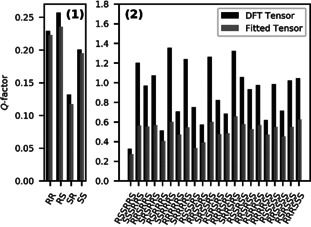
*Q*‐Factors for the different configurations of **1** (left) and **2** (right). The values obtained from predicting the alignment tensor with DFT are shown in black, the ones obtained from the fitted alignment tensor are shown in grey. In all cases, the true configuration is correctly identified by the lowest *Q*‐factor.

In conclusion, we demonstrate how both RDCs and ^13^C RCSAs caused by magnetically induced alignment can be acquired, including a way to efficiently remove systematic errors from chemical shifts, which is crucial for the measurement of the very small RCSAs. These data are accurate enough to successfully distinguish between all different diastereomers of our sample molecules. Second, we show that anisotropic parameters can be fully and reliably predicted using DFT and how this can significantly improve the discriminating power in structure determination. For multiconformer cases, this approach eliminates the need for a common conformer frame, so fewer additional assumptions have to be made. The feasibility of our approach does not directly depend on the degree of alignment, but more on the differences in anisotropic parameters between the diastereomers. This is discussed in more detail in the Supporting Information, Section S4 c. Since even strychnine with only a single aromatic ring can be subjected to the self‐alignment method, we conclude that it is an elegant alternative to alignment media for the determination of the relative configuration of flexible natural products.

## Conflict of interest

The authors declare no conflict of interest.

## Supporting information

As a service to our authors and readers, this journal provides supporting information supplied by the authors. Such materials are peer reviewed and may be re‐organized for online delivery, but are not copy‐edited or typeset. Technical support issues arising from supporting information (other than missing files) should be addressed to the authors.

Supplementary

Supplementary

Supplementary
