# Supplementary material for: Magnetically Induced Alignment of Natural Products for Stereochemical Structure Determination via NMR
Source: Angew Chem Int Ed Engl. 2020 Jun 3;59(37):15860–4. doi: 10.1002/anie.202004881 (PMC7540557; doi:10.1002/anie.202004881)
Supplement: Supplementary file 3 — Supplementary [file ANIE-59-15860-s003.pdf]

Angewandte Chemie
Eine Zeitschrift der Gesellschaft Deutscher Chemiker

Supporting Information

Magnetically Induced Alignment of Natural Products for Stereochemical Structure Determination via NMR

*Niels Karschin, Klaus Wolkenstein, and Christian Griesinger**

anie_202004881_sm_miscellaneous_information.pdf
anie_202004881_sm_data_collection.7z.001
anie_202004881_sm_data_collection.7z.002

Table of Contents

1. Characterization of Gymnochrome G 2
2. Acquisition of Anisotropic Data 4
3. Computational Work / Molecular Modelling 5
4. Data Evaluation 6
5. Instructions for Data Collection 9
6. Author Contributions 9
7. NMR Spectra and Data Tables of Gymnochrome G 10
References 17

1. Characterization of Gymnochrome G

a. Spectroscopic Experimental Procedures. UV spectra were recorded on a Jasco V-630 UV-visible spectrophotometer, ECD spectra were recorded on a Jasco J-810 spectropolarimeter, and IR spectra were measured on a Jasco 4100 FT-IR spectrometer equipped with a Pike Gladi ATR (attenuated total reflection) accessory. 1D and 2D NMR spectra were recorded in MeOH-d$_3$ at 298 K on Bruker Avance III HD and Avance Neo spectrometers at 800 and 900 MHz equipped with TCI CryoProbes. Chemical shifts were referenced using residual solvent peaks (MeOH: δ_H = 3.31 ppm, δ_C = 49.0 ppm). Standard Bruker pulse sequences (zgpr, jmod, hsqcetgpsp.2, hmbcetgpl2nd, hmbcetgpjcl2nd, hsqcdietgp-jcndsisp) were used. ^{1}H–^{13}C HMBC experiments were optimized for $^nJ_{CH}$ = 8 Hz, as well as $^nJ_{CH}$ = 3 Hz for long-range correlations. Long range CH-couplings were measured using *J*-HMBC experiments.[1] The sign of $^2J_{CH}$-couplings was determined using a HSQC-HECADE-type sequence.[2] High-resolution MS spectra were obtained using a Bruker micrOTOF mass spectrometer with electrospray ionization in the negative-ion mode. HPLC was performed on an Agilent 1200 Series system using a Phenomenex Gemini C18 column (250 × 10 mm i.d., 5 µm).

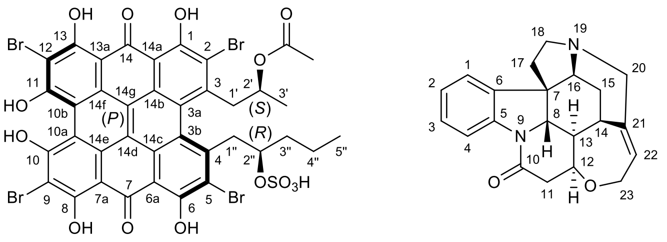

Gymnochrome G (**1**) Strychnine (**2**)

Scheme 1. Constitution, configuration, and numbering of the investigated structures.

b. Animal Material. Two specimen of *H. naresianus* were collected from Shima Spur, Kumano-nada Sea, Japan, from depths of 763 to 852 m. Voucher samples were deposited in the collection of the Systematische Zoologie am Museum für Naturkunde Berlin (ZMB Ech 7415 and ZMB Ech 7416).[3]

c. Extraction and Isolation. Lyophilized *H. naresianus* material (9.0 and 10.1 g) was successively extracted with MeOH/CH_2Cl_2 (1:1), MeOH/H_2O (9:1), MeOH/H_2O (1:1), and distilled water. The methanol-soluble pigments of the MeOH/CH_2Cl_2 extract were combined with the other extracts and subjected to semipreparative HPLC using a linear gradient of acetonitrile/20 mM aqueous ammonium acetate (45:55) to 85% acetonitrile. Fractions were purified and concentrated using solid phase extraction (Bondesil C18, 40 µm). Pigments were washed with water and eluted with MeOH/H_2O (9:1) followed by evaporation of the eluates to dryness to give several anthraquinone and biaryl pigments reported previously,[3] compound **1** and further phenanthroperylene quinones that will be described elsewhere.

d. Molecular Data. Violet solid (2.7 mg); UV (MeOH) λ_{max} (log ε) 220 (4.64), 235 (4.65), 257 (4.59), 300 (4.46), 331 (4.37), 404 (3.86), 496 (4.07), 553 (4.21), 597 (4.50); ECD (MeOH) λ ($\Delta\varepsilon$) 218 (+36.65), 238 (–12.43), 255 (+22.91), 296 (–38.76), 324 (+26.98), 363 (–8.03), 445 (+33.67), 488 (+24.08), 555 (–13.44), 599 (–30.47); IR (ATR) ν_{max} 1570, 1451, 1238, 1128 cm^{-1}; ^{1}H NMR (MeOH-*d*$_3$, 800 MHz), see Table S4; ^{13}C NMR (MeOH-*d*$_3$, 200 MHz), see Table S4; HRESIMS *m/z* 1052.7706 [M–H]$^-$ (calcd for $C_{38}H_{25}Br_4O_{14}S$, 1052.7704), 954 [M–H_3SO_4]$^-$ (calcd for $C_{38}H_{23}Br_4O_{10}$, 954.8030), 525.8836 [M–2H]$^{2-}$ (calcd for 0.5 • $C_{38}H_{24}Br_4O_{14}S$, 525.8816).

e. DP4+ Analysis.[4] The starting geometries were built in Maestro 11.4[5]. For all configurations, the quinoid carbonyl acted as H-bond acceptor for the 1-, 6-, 8-, and 13-OH, the 11-OH group acted as H-bond acceptor for 10-OH, and 12-Br acted as H-bond acceptor for 11-OH. In all following steps this OH conformation was kept fixed to greatly reduce sampling complexity. The sulfate group was built in its deprotonated form. The aromatic ring was built in propeller conformation (*P*). All four combinations of configuration at the two stereogenic centers were generated.

A conformational search was performed with Macromodel 11.8[6] using the MMFF forcefield[7] in vacuum. The method was a Monte-Carlo torsional sampling[8] of all non-terminal rotatable side chain bonds with 100 000 steps, a minimization convergence of 1.0 kJ/mol/Å, and an energy threshold of 21 kJ/mol. The resulting conformers were subjected to a finer minimization with 0.001 kJ/mol/Å convergence, and structures with a maximum atom deviation below 0.5 Å were discarded as duplicates.

All conformers from the ensemble were geometry optimized at the B3LYP[9]/6-31G[10] level of theory with Gaussian09[11], and all conformers below 8.4 kJ/mol of the minimum zero-point corrected (ZPC) free energy were discarded. The shielding constants were calculated at the mPW1PW91[12]/6-31+G(d,p) level of theory using GIAO[13] and an implicit solvent model[14]. The resulting shieldings were then averaged assuming a Boltzmann distribution with the ZPC free energies from the geometry optimization. The shieldings from all side chain H- and C-atoms and the ipso- and ortho-C-atoms of the aromatic system were used to calculate the DP4+ probability. Unassigned methylene protons were assigned to the better-fitting value for each configuration. The resulting DP4+ probabilities were 0.00% ((2'*R*,2''*R*), (2'*R*,2''*S*), (2'*S*,2''*S*)), and 100.00% ((2'*S*,2''*R*))

f. Relative Configuration from *J*-Couplings and ROE Constraints. The chiral aromatic system was assumed to be propeller-(*P*) based on literature data,[15] which was confirmed via ECD calculations (see below). This system could be used as a chirality reference for the side chains. A conformational search suggested that in all relevant conformations the side chains point away from each other (defined by the dihedral around bonds 3-1′ and 4-1″), and therefore the protons at 1′/1″ point towards each other (Fig. S1). This hypothesis was supported by a ROESY spectrum, where correlations between 1′ and 1″ were the only detectable intersidechain contacts. With the conformation around these bonds being known, the diastereotopic protons at 1′ and 1″ could be assigned using ^3J-couplings to the carbon atoms 2, 3a, 3b, and 5. For C-H-arrangements close to antiperiplanar larger *J*-couplings are expected than for synperiplanar arrangements. E.g., Proton H1a″ has a larger coupling to C5 than to C3b, while H1b″ shows the opposite trend. Therefore, H1a″ has to be the proton pointing inward (pro-*R*), while H1b″ is the proton pointing outward (pro-*S*) (Fig. S1). The same argument applies to H1′. Having established the assignment of these methylene protons, the dihedral conformation of bonds 1′-2′ and 1″-2″ can be examined. The relevant couplings are $^3J_{H1H2}$, $^3J_{H1C3}$, and $^2J_{H1C2}$ (with primes added accordingly). ^3J is large for an antiperiplanar

arrangement and small for a synclinal arrangement of the coupling nuclei, while $^{2}J_{CH}$ couplings are most useful if there is an electronegative substituent X on the carbon atom (which there is in both side chains). In that case, the apparent $^{2}J_{CH}$ is large if the proton and X have a synclinal arrangement and small if they have an antiperiplanar arrangement. A review of these relations can be readily found in literature.[16] Following these arguments, there is only one possible arrangement of substituents that is in agreement with the measured couplings, and the configuration of the stereogenic centers can directly be determined. This can be most easily seen as a Newman projection (Fig. S2). All relevant couplings are shown in Table S5.

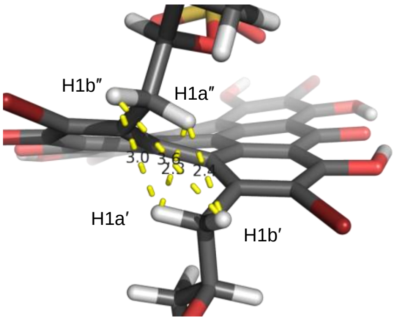

Figure S1. Visible ROE contacts and their respective distances in a conformer of gymnochrome G.

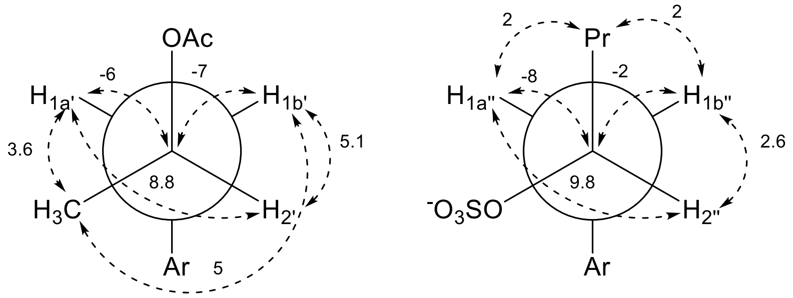

Figure S2. Newman projections along C2′-C1′ (left) and and C2″-C1″ (right) with measured *J*-couplings in Hz.

g. Absolute Configuration from ECD. The configuration of the aromatic system was confirmed to be propeller-(*P*) by ECD calculations. The conformations generated to analyze the anisotropic data were used as a starting point (see section S3). We performed an excited state calculation with 100 states using time-domain DFT using three different functionals PBE0[17], B3LYP, and CAM-B3LYP[18], and the pcseg-1 basis set. It is recommended in the literature to use different types of functionals (with and without long-range corrections, and different fractions of exact exchange).[19] To reduce computational demand, only conformers with a free energy of less than 11.4 kJ/mol above the ground state were considered (i.e., ~1% population at 298 K). The first calculations with the PBE0 functional revealed that the ECD is largely dominated by the aromatic system and has very little dependence of the side chain conformation and configuration. Therefore, the calculations with B3LYP and CAM-B3LYP were only performed on the correct configuration (2'*S*,2''*R*). The generation of spectra and Boltzmann averaging with ZPC free energies was done using SpecDis 1.71.[20] We also used the functionality in SpecDis to calculate similarity factors, which are a measure of similarity between an experimental and a calculated spectrum.[20a, 21] In this procedure, the UV correction, the bandwidth σ, and the scaling factor are optimized automatically (see Table S1). The UV correction is an empirical shift along the wavelength axis of the calculated spectrum which accounts for systematic errors in transition energies.[19] The bandwidth σ is simply the standard deviation of the Gaussian distribution used to transform a stick spectrum of transition energies into a continuous spectrum comparable to the experiment. The scaling factor scales the intensity of the calculated spectra to match the experiment. This is necessary as absolute intensities are generally inaccurate in ECD calculations. The similarity factors, which range from 0 to 1, are calculated for both enantiomers. Generally, the spectra calculated without long-range corrections (B3LYP and PBE0, Figure S3) show much better agreement with the experiment than CAM-B3LYP. But for all cases both the visual comparison as well as the similarity factors (Table S1) show clearly that our original assumption for the configuration (*P*) was correct.

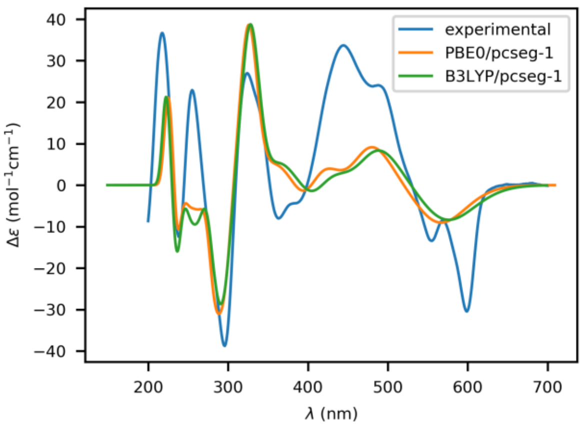

Figure S3. Experimental ECD curve compared to calculated ECD curves at (PBE0, B3LYP)/pcseg-1 level of theory.

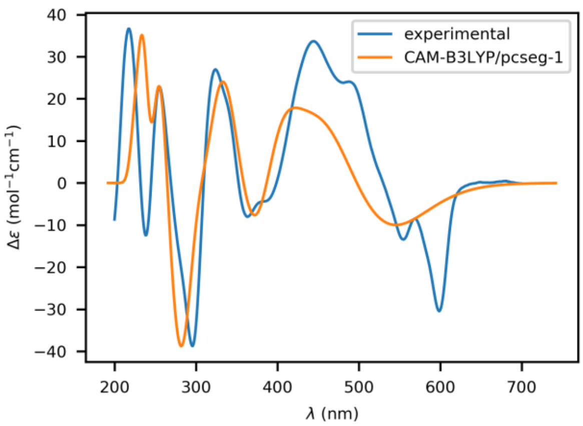

Figure S4. Experimental ECD curve compared to calculated ECD curves at CAM-B3LYP/pcseg-1 level of theory.

Table S1. Comparison criteria of calculated ECD data of gymnochrome G.

Functional	PBE0	B3LYP	CAM-B3LYP
Scaling factor	0.2508	0.2574	0.4163
Bandwidth σ (eV)	0.17	0.17	0.3
UV shift (nm)	9	-1	43
Similarity factor	0.8025	0.8130	0.6905
Similarity factor (enantiomer)	0.0448	0.0686	0.0504

2. Acquisition of Anisotropic Data

a. Sample Preparation. For **1**, 2.0 mg of the dry material from purification was dissolved in ca. 150µL MeOH-d_3 and transferred into a 3 mm NMR tube. For **2**, a 200 mM solution of strychnine (Sigma-Aldrich) in $CDCl_3$ with ca. 1% v/v TMS was washed twice 1:1 with 250 g/L K_2CO_3 (aq) to remove traces of chloroform degradation products. The solution was then dried over K_2CO_3 (s), transferred into a 5 mm NMR tube, and degassed using 5 freeze-pump-thaw cycles. Finally, the tube was flame-sealed under vacuum.

b. Spectroscopic Experimental Procedures. 1D and 2D NMR spectra were recorded on Bruker Avance III HD or Bruker Avance Neo spectrometers at fields between 9.4 T and 22.3 T (proton frequencies of 400, 600, 700, 800, 900, and 950 MHz) using inverse cryogenically cooled (QCI/TCI) probes (600-950 MHz) and a room temperature inverse (QXI) probe (400 MHz). Chemical shifts were referenced using residual solvent peaks (δ_H = 3.31 ppm, δ_C = 49.0 ppm) for **1** and TMS for **2**. The sample temperature was set to 298 K. For **1**, the temperature control was cross-checked and fine-tuned using a 99.8% MeOD-d_4 reference sample (Sigma-Aldrich).[22] For **2**, the chemical shift of the residual water peak (2.397 ppm @ 298 K, −23 ppb/K temperature shift) served as internal temperature standard. Proton 1D spectra were acquired using simple pulse-acquire sequences. For **1**, low power presaturation was added for solvent suppression. Spin-echo based solvent suppression (WATERGATE etc.) is less suitable as it introduces phase errors in the multiplet structure due to homonuclear coupling evolution. Carbon 1D spectra were acquired using *J*-modulated echo sequence (Bruker standard sequence jmod) with 4 – 5 kHz proton decoupling. For **2**, HSQC spectra were acquired using a perfect-CLIP-HSQC sequence.[23]

c. Data Processing. For data processing, zero-filling to 1024k (2^{20}) points (1D spectra) and 64k (2^{16}) points (HSQC) was applied in the direct dimension. For the low-sensitivity carbon-based experiments (*J*-mod/HSQC), 0.3 Hz line-broadening was applied before Fourier transformation. Peak picking and multiplet analysis were then done in MestreNova 11. For **1**, J_{HH} couplings were taken from the proton 1D, and peaks showing overlap were discarded. For **2**, all couplings were extracted from the HSQC. The slices in the proton dimension containing the relevant peaks were extracted for this purpose. Any HSQC peaks showing overlap with long range correlations of neighboring peaks were discarded. This was the case for the atom positions 1, 2, and 3. Since all RDCs were much smaller than the corresponding scalar coupling *J*, the sign of the RDC follows from the sign of *J*. All $^2J_{HH}$ were assumed to be negative, all others to be positive.

d. Elimination of Chemical Shift Errors. For this purpose, the temperature and time/degradation dependence of the shifts had to be determined. For temperature, *J*-mod spectra at different temperatures, but equal field were acquired (**1**: 297 K, 298 K, 299 K @ 800 MHz, **2**: 298 K, 301 K @ 900 MHz). For each resonance, a linear fit of the form $\delta = \delta_0 + T\delta_T$ was performed with δ_0 and δ_T as fit parameters; all resulting δ_T were arrayed as a vector $\vec{\delta}_T$. For degradation, *J*-mod spectra at different time points, but equal fields were acquired. We acquired three pairs of spectra for **1** and two pairs of spectra for **2** with time differences between one and three months to verify the reproducibility of this approach. For practical application we recommend acquiring the data as quickly as possible, and to repeat the measurement of the first field point at the end of the measurement series. This way the degradation effect during the measurement series is captured, requiring only one additional measurement and no extra waiting time. To give the reader an idea of the magnitude of these effects, the root mean square of the temperature dependence of the carbon chemical shifts was 3.3 ppb/K for **1** and 5.9 ppb/K for **2**, while for the degradation it was 3.7 ppb for **1** and 0.4 ppb for **2**. The chemical shift differences of all resonances for such a pair of spectra are then arrayed as a vector $\vec{\delta}_{\mathrm{degrad}}$. In the case of **2**, small changes in referencing were also a source of error, supposedly due to small temperature and degradation effects on the reference shift (TMS). Since all resonances are shifted by an equal amount by the referencing, the elements of the vector $\vec{\delta}_{\mathrm{ref}}$ representing this change are equal (e.g., 1). Note that only the direction of these perturbation vectors is relevant, but not their magnitude. These perturbation vectors are now arrayed into an $m \times n$ matrix $\boldsymbol{D}$, where is m the number of resonances and n is the number of perturbation vectors, and a QR decomposition of this matrix is performed. The resulting orthogonal matrix $\boldsymbol{Q}$ can be used to perform an orthonormal transformation of the chemical shift space: the chemical shifts from a given spectrum are arrayed into a vector $\vec{\delta}$, which is then transformed into $\vec{\delta}^* = \boldsymbol{Q}^{\mathrm{T}} \cdot \vec{\delta}$. The first n elements of $\vec{\delta}^*$ are affected by the systematic errors and have to be discarded, while the remaining ones can be used as structural constraints in the following process.

e. Determination of Anisotropic Components. To determine RCSAs, the corrected chemical shifts were expressed as peak position in Hz, and they obey the following relation:

$$\nu_{\mathrm{meas}} = \delta_{\mathrm{iso}}\nu_{\mathrm{ref}} + \delta_{\mathrm{aniso}}\nu_{\mathrm{ref}}B_0^2 = \delta_{\mathrm{iso}}\gamma B_0 + \delta_{\mathrm{aniso}}\gamma B_0^3 \,. \quad (1)$$

In this context, the unknown parameters δ_{iso} and δ_{aniso} correspond to the isotropic chemical shift in ppm and to the desired RCSA in ppm T^{-2}, respectively. As they both appear in linear form in Equation (1), the least-squares solution to a given set of (ν_{meas}, B_0) data can be determined easily and deterministically. Similarly, the measured coupling $\Delta\nu_{\mathrm{meas}}$ obeys the following relation as a function of the field:

$$\Delta\nu_{\mathrm{meas}} = \Delta\nu_{\mathrm{iso}} + \Delta\nu_{\mathrm{aniso}} B_0^2 \,. \tag{2}$$

Here, $\Delta\nu_{\mathrm{iso}}$ and $\Delta\nu_{\mathrm{aniso}}$ correspond to the *J*-coupling in Hz and to the RDC in Hz T^{-2}, respectively, and can again be determined as least-squares solution to a given set of ($\Delta\nu_{\mathrm{meas}}$, B_0). As mentioned above, δ_{aniso} and $\Delta\nu_{\mathrm{aniso}}$ have units of ppm T^{-2} and Hz T^{-2}, respectively, but for all subsequent data interpretation they were converted into frequency units by evaluating the anisotropic components of Equations (1) and (2) at $B_0 = 23.49$ T (1 GHz proton frequency). While it is a somewhat arbitrary choice, it enables the simultaneous interpretation of RDCs and RCSAs. We give them both the same weight since at this field the one bond CH-RDCs are in size between aliphatic and aromatic ^{13}C-RCSAs. We excluded couplings from the evaluation that showed obvious peak distortions (see above). For strychnine, $^{1}D_{\mathrm{CH}}$ - couplings from the two protons of a methylene group were evaluated as their mean value to remove the need for diastereotopic assignment. A given geminal $^{2}J_{\mathrm{HH}}$ + $^{2}D_{\mathrm{HH}}$ -coupling was determined individually from each proton peak and averaged.

f. Determination of Weighting Factors. Since the alignment in strychnine is about a factor of 5 smaller than for gymnochrome G, the data are more sensitive to errors, and different RDCs/RCSAs may have different errors associated to them. We therefore devised a way to determine weighting factors to give a lower importance to data points with higher uncertainty. In practice, this was most important for RDCs, but we applied it to both RDCs and RCSAs. Determining weighting factors for both types of parameters is a general way to make simultaneous evaluation possible. In the following, we illustrate the procedure on the example of RDCs.

All data points are affected by a random error, and the uncertainty of the anisotropic component is proportional to this error. In principle, the error (standard deviation) of each field-series of couplings can be estimated by calculating the RMSD s_i of the fit to Eq. (2):

$$s_i = \mathrm{RMS}\left(\Delta\nu_{\mathrm{meas}}^{j} - \Delta\nu_{\mathrm{model}}^{j}\right), \tag{3}$$

where the indices i and j refer to the coupling (atom pair) and the field, respectively. Since there are only six data points (fields) along j, there is a significant uncertainty associated with this. If this RMSD is used as a weighting factor to the corresponding RDC, data points that by chance have a very low RMSD will be overvalued. However, since some few couplings are affected by stronger peak distortion, there was the need to devalue them relative to the more accurate RDCs, i.e., determine individual weighting factors. We made the following assumption: All couplings are equally affected by the same (random) base error. Some few couplings are then additionally affected by significant individual errors due to peak distortions etc. If there were no individual errors, the base error could be estimated by taking the RMS of the RMSD of all field-series fits. Some of these field-series are affected by additional errors, and lead to outliers in their corresponding RMSD, which greatly affects this RMS of RMSD. To cope with that, we estimate the base error s_{base} by calculating the root median square instead:

$$s_{\mathrm{base}} = \sqrt{\mathrm{median}(s_i^2)} \,. \tag{4}$$

Now, we estimate the individual error as the RMSD of the field-series fit and calculate the total error as RMS of base and individual error. We use the inverse of the error as weight w_i:

$$w_i = \frac{1}{\sqrt{s_i + s_{\mathrm{base}}}} \,. \tag{5}$$

This enables a smooth transition between small and large weights, prevents the overvaluation of data points, and enables to give smaller weights to data points with large error.

3. Computational Work / Molecular Modelling

a. General Procedure. For **1**, the conformer geometries from the DP4+ calculations at the MMFF level of theory were used as starting points. For **2**, starting structures were built in Maestro 11.4. The multiple fused and bridged cycles of **2** allow only a subset of the theoretically possible 32 diastereomers, and we were able to build 22 configurations. Although some of these have strongly distorted carbon binding geometries and could be discarded as they are unlikely to be stable, we purposefully left them in the analysis as this further demonstrates the discriminating power of the method. We cross-checked the resulting geometries with the structures of Bifulco et al.,[24] who conducted a thorough study of the possible diastereomers of strychnine. We optimized all geometries at the B3LYP/pcseg-1[25] level of theory, including vibrational frequencies for **1** to get zero-point corrected (ZPC) free energies. We calculated NMR shieldings and the magnetic susceptibility tensors at the B3LYP/pcSseg-1 (**1**) and B3LYP/pcSseg-2[26] (**2**) level of theory using GIAO[13] and an implicit solvent model[14]. All DFT calculations were performed in Gaussian09.[11]

b. Convergence Problems for Susceptibility Calculations. During the first attempts to predict magnetic susceptibilities, we encountered severe basis set convergence problems, which were most prominent for large Pople-type basis sets. We investigated this by calculating shieldings and susceptibilities for strychnine using 57 different basis sets, from STO-3G to 6-311++G(3df,3pd). For relatively small basis sets (e.g., 6-31G, 6-31G(d)) the agreement of the calculated alignment (i.e., the susceptibility anisotropy) with the experimental data was good, but with larger basis sets it became increasingly worse. This was most extreme for basis sets with diffuse functions (i.e., functions with small exponents decaying slowly with distance), which points to numerical artifacts due to near degeneracy of some basis set functions. It is noteworthy that these numerical artifacts had a significant effect only on the susceptibilities, but not on geometries or nuclear shielding tensors. These numerical artifacts could be avoided by using a finer than default integration grid using the Gaussian "UltraFine" keyword. As an additional measure, we decided to switch to a more balanced family of basis sets with a clear hierarchy. We tested Jensen's polarization-consistent segmented basis sets as well as Karlsruhe (def2) basis sets[27] on strychnine, which yielded very similar results. We finally chose to do all subsequent calculations with Jensen-type basis sets as they were specifically designed for DFT calculations and provide a subfamily optimized for nuclear properties (pcSseg-n).

4. Data Evaluation

a. Calculation of Anisotropic Data. As mentioned in the main article, the anisotropic parameters obey the following relations:

$$\Delta\nu_{\mathrm{RDC}} = -\frac{3\gamma_I\gamma_S\mu_0\hbar}{8\pi^2\left\|\vec{R}\right\|^5}\left(\vec{R}^{\mathrm{T}}\boldsymbol{A}\,\vec{R}\right) \tag{6}$$

$$\Delta\delta_{\mathrm{RCSA}} = \mathrm{tr}(\boldsymbol{A}\boldsymbol{\delta})\,, \tag{7}$$

where γ are the gyromagnetic ratios of the coupling nuclei and $\vec{R}$ the internuclear vector between them, $\boldsymbol{\delta}$ is the chemical shift tensor of the nucleus in question, and $\boldsymbol{A}$ is the alignment tensor. The alignment tensor depends on the magnetic susceptibility tensor $\boldsymbol{\chi}$:

$$\boldsymbol{A} = \frac{(\boldsymbol{\chi} - \chi_{\mathrm{iso}}\mathbf{1})\,B_0^2}{(15k_{\mathrm{B}}T)}. \tag{8}$$

Since all quantities are either constants ($\gamma, \mu_0, \hbar, k_{\mathrm{B}}$), known experimental parameters (T, B_0), or molecular properties that can be calculated via DFT ($\vec{R}, \boldsymbol{\chi}, \boldsymbol{\delta}$), theoretical RDCs and RCSAs can be calculated by simply inserting all constants and parameters into Equations (6) and (7). For **1**, averaging of the calculated anisotropic data is done over all conformers using ZPC free energies $G_{\mathrm{ZPC},k}$ using the following Boltzmann populations p_k:

$$p_k = \frac{\exp(-G_{\mathrm{ZPC},k}/k_{\mathrm{B}}T)}{\sum_k \exp(-G_{\mathrm{ZPC},k}/k_{\mathrm{B}}T)}, \tag{9}$$

where the index k refers to the conformation. For the fit of the alignment tensor, Equations (6) and (7) have to be brought into a form where the linearity of the components of $\boldsymbol{A}$ is apparent. First, it is helpful to realize that both equations have the exact same form:

$$x = \mathrm{tr}(\boldsymbol{A}\boldsymbol{M})\,. \tag{10}$$

For $x = \Delta\nu_{\mathrm{RDC}}$ it is $\boldsymbol{M} = -(3\gamma_I\gamma_S\mu_0\hbar)/(8\pi^2\left\|\vec{R}\right\|^5)\,\vec{R}\,\vec{R}^{\mathrm{T}}$, while for $x = \Delta\delta_{\mathrm{RCSA}}$ it is obviously $\boldsymbol{M} = \boldsymbol{\delta}$. This can be expanded into

$$x = \sum_{i,j=x,y,z} A_{ij}M_{ij} \tag{11}$$

and rearranged, by using the fact that $\boldsymbol{A}$ and $\boldsymbol{M}$ are real symmetric:

$$x = \begin{pmatrix} \frac{1}{3}(A_{xx}+A_{yy}+A_{zz})(M_{xx}+M_{yy}+M_{zz}) + \frac{1}{6}(2A_{zz}-A_{yy}-A_{xx})(2M_{zz}-M_{yy}-M_{xx}) + \frac{1}{2}(A_{xx}-A_{yy})(M_{xx}-M_{yy}) \\ +2A_{xy}M_{xy} + 2A_{xz}M_{xz} + 2A_{yz}M_{yz} \end{pmatrix}. \tag{12}$$

The reason that the products of diagonal elements are rearranged in this way is that now the first summand can be discarded since $\boldsymbol{A}$ is traceless, i.e., $A_{xx} + A_{yy} + A_{zz} = 0$. What is left is a linear equation of five independent components of $\boldsymbol{A}$. This becomes an (ideally overdetermined) system of linear equations if all data points x (RDC/RCSA) with their individual $\boldsymbol{M}$ are taken together, and the least-squares solution of this system yields the fitted alignment tensor. This fitted alignment tensor can then in return be used to back-calculate the anisotropic data.

For **1**, there is the additional complication of multiple conformers. While in principle each conformer has its individual alignment tensor, this would increase the number of free variables beyond feasibility, but it is also not necessary. This is because the main source of alignment (i.e., the aromatic system) has no conformational freedom. Therefore, we can ignore these differences and apply a single tensor approximation. To do so, we use a common frame defined by the aromatic system for all conformers. In practice this is done by defining the vectors $\vec{v}_1 = \overrightarrow{\mathrm{C12}} - \overrightarrow{\mathrm{C5}}$, $\vec{v}_2 = \overrightarrow{\mathrm{C9}} - \overrightarrow{\mathrm{C2}}$, and $\vec{v}_3 = \vec{v}_2 \times \vec{v}_3$, orthonormalize them, and use them as base vectors for a common Cartesian coordinate system. In this common frame, we calculate the components of the matrices $\boldsymbol{M}_k^{\mathrm{cf}}$ and average them, again with the ZPC free energies. The alignment tensor fit is done using these averaged $\boldsymbol{M}$, so the fitting Equation (10) adapts to the following:

$$x = \mathrm{tr}\left(\boldsymbol{A} \sum_k p_k \boldsymbol{M}_k^{\mathrm{cf}}\right). \tag{13}$$

Note that it would be a mistake to average over coordinate vectors as they contribute quadratically to the RDC coefficient matrix $\boldsymbol{M}_k^{\mathrm{cf}}$.

b. Comparison of Experimental and Calculated Data. Both ways of determining the alignment tensor, "fitted" versus "predicted", yield a set of calculated data for each configuration, which is compared with the experimental data to identify the best match. This comparison is done in the same way for both back-calculated and DFT-predicted data sets. As primary criterion for the agreement of the calculated and experimental data we use the Q-factor,[28] which is the RMSD between the two, scaled by the RMS of the experimental data:

$$Q = \sqrt{\frac{\sum_i w_i^2 \left(x_{\mathrm{exp},i} - x_{\mathrm{calc},i}\right)^2}{\sum_i w_i^2 x_{\mathrm{exp},i}^2}}, \tag{14}$$

where x refers to any type of data point. The configuration whose calculated data gives the lowest Q-factor is assumed to be the correct configuration. The Q-factors can be found in Tables S2 and S3. However, this approach gives no quantitative estimate for the confidence of the result since differences in Q-factors cannot be directly interpreted as a measure for the confidence. We use a bootstrapping test to examine the statistical properties of the data and get an insight on the confidence of our result.[29] Bootstrapping has the advantage that no assumptions about source, size, and distribution of error have to be made. In this procedure, resampled data sets of the same original size are generated by drawing randomly with replacement from the experimental data. These resampled sets are then each subjected to the same analysis as the original data set. The DFT-predicted data has to be simply re-matched to the sampled experimental data points. However, the procedure of fitting the alignment tensor and back-calculating the data has to be repeated for each resample. By repeating this resampling many times (in our case 10^6) one gets a distribution of any quantity that results from the data evaluation. As mentioned before, we assume the correct configuration to be the one with the lowest Q-factor, so we did this for each resample. The fraction of resamples that yield a certain configuration is then interpreted as confidence in this result. These confidences are shown in Figure S5, as well as in Tables S2 and S3. We do not use Q_{CSA}[30] in this work which was designed for external alignment with RCSAs of several Hz, where the error is largely independent of the value of the RCSA in Hz. Here, due to the smallness of the effects, the error of the RCSA measurement is mainly determined by the size of the RCSA. Therefore, the underlying assumption that makes the Q_{RCSA} parameter more distinctive for external alignment is not applicable for the self-alignment presented here.

c. A Note on Feasability. The feasibility of our approach does not directly depend on the degree of alignment, but more on the differences in anisotropic parameters between the diastereomers. As these can be accurately predicted, the feasibility for any given molecule can be evaluated with inexpensive DFT calculations before investing expensive NMR time. The molecule strychnine is certainly near the limit of what we assume to be feasible at this point; we have calculated expected anisotropic parameters for various non-aromatic compounds (e.g., sugars) and they are typically about an order of magnitude smaller than for an aromatic compound such as strychnine. But also the geometrical differences of a set of diastereomers obviously affect how well they can be discriminated. Lastly, the availability of high magnetic fields plays an important role. Since the magnitude of anisotropic effects scales with the square of the field while the measuring accuracy is (in first approximation) constant, having very high fields such as 1.2 GHz becoming available right now greatly enhances the feasibility of our approach. To be more quantitative, the weighted RMSD $s_{\mathrm{exp,calc}}$ of the experimental and calculated anisotropic data (RDCs and ^{13}C RCSAs) for strychnine

$$s_{\mathrm{exp,calc}} = \sqrt{\frac{\sum_i w_i^2 \left(x_{\mathrm{exp},i} - x_{\mathrm{calc},i}\right)^2}{\sum_i w_i^2}} \tag{15}$$

is $s_{\mathrm{exp,calc}} = 0.042$ Hz. This can be taken as an estimate of the current measurement accuracy. The RMSD $s_{a,b}$ of calculated anisotropic data for two different configurations a and b of a molecule should therefore be larger than this for discrimination to be possible:

$$s_{a,b} = \sqrt{\frac{\sum_i \left(x_{\mathrm{calc},i}^a - x_{\mathrm{calc},i}^b\right)^2}{\sum_i 1}} > 0.042\ \mathrm{Hz}. \tag{16}$$

Table S2. *Q*-factors and confidences *p* for strychnine (**2**) for both approaches. A confidence of 0 is equivalent to it being smaller than 1e-6.

Configuration	*Q* (DFT)	*Q* (fitted)	*p* (DFT)	*p* (fitted)
RSSRRS	0.326	0.273	9.92e-01	7.66e-01
SSSRRS	1.200	0.563	0.00e+00	2.19e-03
RRSRRS	0.969	0.552	0.00e+00	6.75e-03
SRSRRS	1.073	0.569	0.00e+00	6.97e-04
RSRRRS	0.512	0.407	5.00e-03	1.55e-02
SSRRRS	1.354	0.602	0.00e+00	6.73e-04
RRRRRS	0.705	0.470	0.00e+00	4.30e-03
SRRRRS	1.239	0.545	0.00e+00	6.47e-04
RSSSRS	0.751	0.334	0.00e+00	1.52e-01
RRSSRS	0.573	0.392	1.68e-03	3.15e-02
SRSSRS	1.263	0.602	0.00e+00	9.10e-05
RSRSRS	0.822	0.475	0.00e+00	1.13e-03
RRRSRS	0.684	0.485	2.90e-05	1.93e-03
SRRSRS	1.323	0.658	0.00e+00	2.10e-05
RSSRSS	1.057	0.577	0.00e+00	4.37e-03
RRSRSS	0.931	0.525	0.00e+00	7.30e-03
RSRRSS	0.976	0.568	0.00e+00	3.21e-04
RRRRSS	0.620	0.470	3.10e-05	1.68e-03
RSSSSS	0.986	0.548	0.00e+00	2.03e-04
RRSSSS	0.713	0.452	8.57e-04	8.44e-04
RSRSSS	1.020	0.550	0.00e+00	1.43e-03
RRRSSS	1.044	0.626	0.00e+00	9.00e-06

Table S3. *Q*-factors and confidences *p* for gymnochrome G (**1**) for both approaches.

Configuration	*Q* (DFT)	*Q* (fitted)	*p* (DFT)	*p* (fitted)
RR	0.229	0.223	7.46e-04	3.72e-03
RS	0.257	0.235	1.23e-04	9.20e-05
SR	0.132	0.118	8.80e-01	9.57e-01
SS	0.201	0.195	1.19e-01	3.88e-02

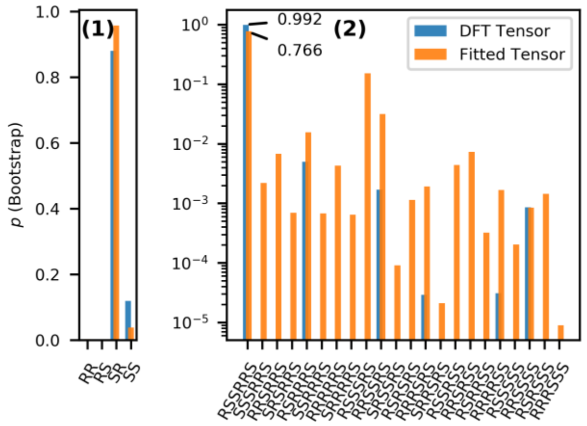

Figure S5: Fraction of bootstrap resamples *p* that point to a certain configuration (i.e., that configuration has the lowest *Q*-factor for that resample) for the two different apporaches of data evaluation. Mind the logarithmic scale in the case of **2**.

5. Instructions for Data Collection

All numerical data is published in digital form instead of tables in this SI for improved accessibility. There are three subfolders: computation_logs contains all data concerning the molecular modelling, anisotropic_datasets contains all raw experimental data, and intermediate_results contains fitted and calculated RDCs/RCSAs and the data needed for error compensation. The folder computation_logs contains several subfolders with self-explanatory folder names. Each of these folders contains one or two subfolders corresponding to the two investigated molecules.

a. Computation: Gaussian. The geometry optimizations, the frequency calculations, the NMR calculations, and the ECD calculations were done in Gaussian. The folders contain Gaussian logfiles and Gaussian comfiles. The comfiles do not include the geometry since it was always read from a previous checkpoint file. However, the logfiles always contain the geometry information. The filenames refer to the molecular structure. For **1**, it is of the form "g8_xx_stateiiii.com/log", where xx indicates the configuration and iiii the conformation number. For **2**, it is "strychnine_ii.com/log", where ii denotes the number of the configuration. 01 is the correct configuration, the others are in somewhat arbitrary order. The order can be found in the figures in the main text or in the file confs.txt in the folder with the starting geometries.

b. Computation: Conformational Search. The starting geometries are stored in the appropriate folder. For **2**, they are stored in xyz format. For **1**, they are stored in mol2 format. For the conformational search of **1**, Macromodel logfiles and comfiles for the conformational search as well as the following finer minimization are provided. Input and output structures in Maestro's own format are provided as well. The resulting conformers are provided in one file per configuration in mol2 format.

c. Raw Data: Chemical Shifts. Chemical shifts are provided in text files with tab separation and a header line for the identification of columns. They are simply copied peak tables from MestreNova 11.0 and are also self-explanatory. The field is given in the filename. Files named T1, T2, or T3 contain temperature dependent shifts. For **1**, all data was acquired twice for each field, and the degradation dependence was determined from those pairs (e.g., 600_C_1.txt and 600_C_2.txt). For **2**, the pairs for the determination of the degradation vector were stored separately in files starting with deg or deh. In both cases, mnova files containing the spectral data are also included.

d. Raw Data: Couplings. Couplings for **1** were extracted from 1D proton spectra, which was not possible to automate since multiplets would overlap differently depending on the field. The suitable multiplet components for the determination of the coupling were therefore chosen manually and the couplings deposited in couplings.csv. For **2**, couplings were extracted from CLIP-HSQC traces. All traces are provided as mnova files, separated by field (e.g., 400_Js_strychnine.mnova). The extracted couplings are saved in tab-separated text files (e.g., 400_J.txt).

e. Intermediate Data. In the folder intermediate_results the following data are provided: carbon resonance assignment, chemical shift error vectors $\vec{\delta}_{\mathrm{T}}$ and $\vec{\delta}_{\mathrm{degrad}}$, the transformation matrix $\boldsymbol{Q}$, and the fitted RDCs and RCSAs with the fit residuals and the weighting factors (for **2**). All data are provided as tab-separated text files with extensive comments.

6. Author Contributions

NK designed the project, measured and evaluated the data, and wrote the original draft. KW isolated gymnochrome G and determined its constitution. CG supervised the work and administered the project. All authors discussed the work and contributed to writing.

7. NMR Spectra and Data Tables of Gymnochrome G

Table S4. NMR spectroscopic data for gymnochrome G (**1**).[a]

position	δ_C (mult)	δ_H, mult. (splitting in Hz)	HMBC
7	185.83 (C_q)		
14	185.70 (C_q)		
11	170.31 (C_q)		
10	170.09 (C_q)		
13	166.43 (C_q)		
8	166.37 (C_q)		
6	160.92 (C_q)		
1	160.70 (C_q)		
4	145.00 (C_q)		
3	142.53 (C_q)		
	127.65 [b] (C_q)		
	127.55 [b] (C_q)		
	126.60 [b] (C_q)		
	126.55 [b] (C_q)		
3b	123.79 (C_q)		
3a	123.47 (C_q)		
	122.93 [b] (C_q)		
	122.92 [b] (C_q)		
	119.46 [b] (C_q)		
	119.38 [b] (C_q)		
5	116.82 (C_q)		
2	116.52 (C_q)		
14a	110.85 (C_q)		
6a	110.84 (C_q)		
13a	104.25 (C_q)		
7a	104.16 (C_q)		
9	103.80 (C_q)		
12	103.77 (C_q)		
1′	44.51 (CH_2)	4.09 dd (13.5; 5.1), 3.82 dd (13.4; 8.8)	2, 3, 3a, 2′
2′	71.15 (CH)	4.79 m	O*C*OCH_3
2′-O*C*OCH_3	171.53 (C_q)		
2′-OCO*CH_3*	20.83 (CH_3)	1.81 s	O*C*OCH_3
3′	18.59 (CH_3)	0.15 d (6.2)	1′, 2′
1″	44.06 (CH_2)	3.92 dd (14.3; 2.6), 3.86 dd (14.2; 9.6)	3b, 4, 5, 2″
2″	79.95 (CH)	4.27 m	
3″	38.72 (CH_2)	1.48 m, 1.64 m	2″, 5″
4″	18.87 (CH_2)	1.42 m, 1.47 m	5″
5″	14.42 (CH_3)	0.87 t (7.3)	3″, 4″
1-OH		14.94 s	1, 2, 14a, 14
6-OH		14.92 s	5, 6, 6a, 7
8-OH		15.47 s	7a, 8, 9, 10, 7
13-OH		15.44 s	11, 12, 13, 13a, 14

[a] Spectra were recorded in MeOH-d_3 at 800 MHz proton frequency.

[b] These signals show no correlation to protons and could not be individually assigned. They belong to the carbon atoms 10a, 10b, 14b, 14c, 14d, 14e, 14f, and 14g, which can be deduced by comparison to known gymnochromes.[31] These resonances were therefore not used in any data analysis.

Table S5. ^{13}C-^{1}H and ^{1}H-^{1}H coupling constants for gymnochrome G (**1**).

Propyl acetate side chain		Pentyl sulfate side chain	
J-HMBC correlation	*J* in Hz	*J*-HMBC correlation	*J* in Hz
$^{3}J_{C3a–H1'a}$	3	$^{3}J_{C3b–H1''a}$	4
$^{3}J_{C3a–H1'b}$	5	$^{3}J_{C3b–H1''b}$	5
$^{3}J_{C2–H1'a}$	8	$^{3}J_{C5–H1''a}$	8
$^{3}J_{C2–H1'b}$	4	$^{3}J_{C5–H1''b}$	4
$^{3}J_{H2'–H1'a}$	8.8	$^{3}J_{H2''–H1''a}$	9.8
$^{3}J_{H2'–H1'b}$	5.1	$^{3}J_{H2''–H1''b}$	2.6
$^{3}J_{C3–H2'}$	2	$^{3}J_{C4–H2''}$	3
$^{3}J_{C3'–H1'a}$	4	$^{3}J_{C3''–H1''a}$	2
$^{3}J_{C3'–H1'b}$	5	$^{3}J_{C3''–H1''b}$	2
$^{2}J_{C2'–H1'a}$	-6	$^{2}J_{C2''–H1''a}$	-8
$^{2}J_{C2'–H1'b}$	-7	$^{2}J_{C2''–H1''b}$	-2

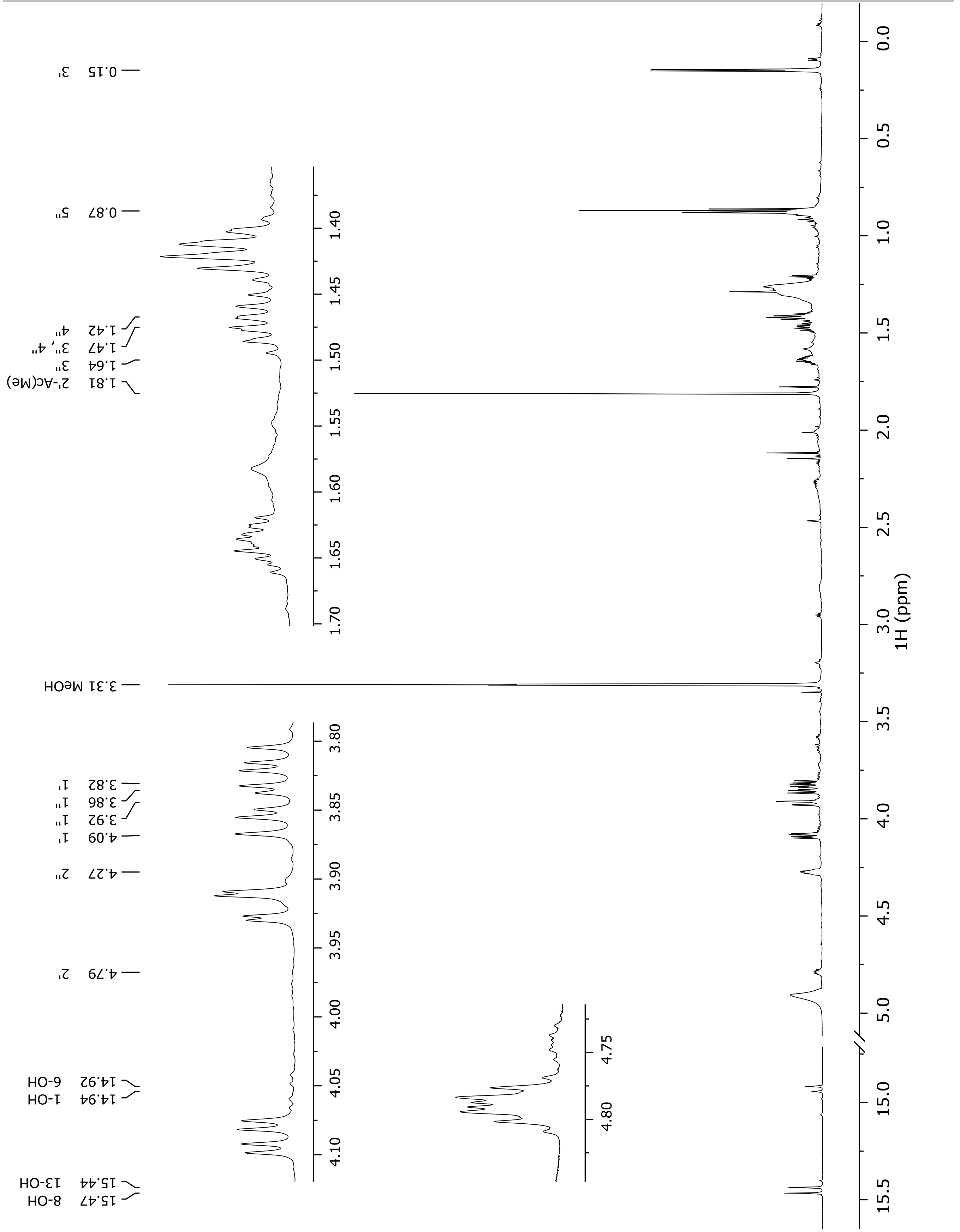

Figure S6. 800MHz ^{1}H NMR spectrum of gymnochrome G.

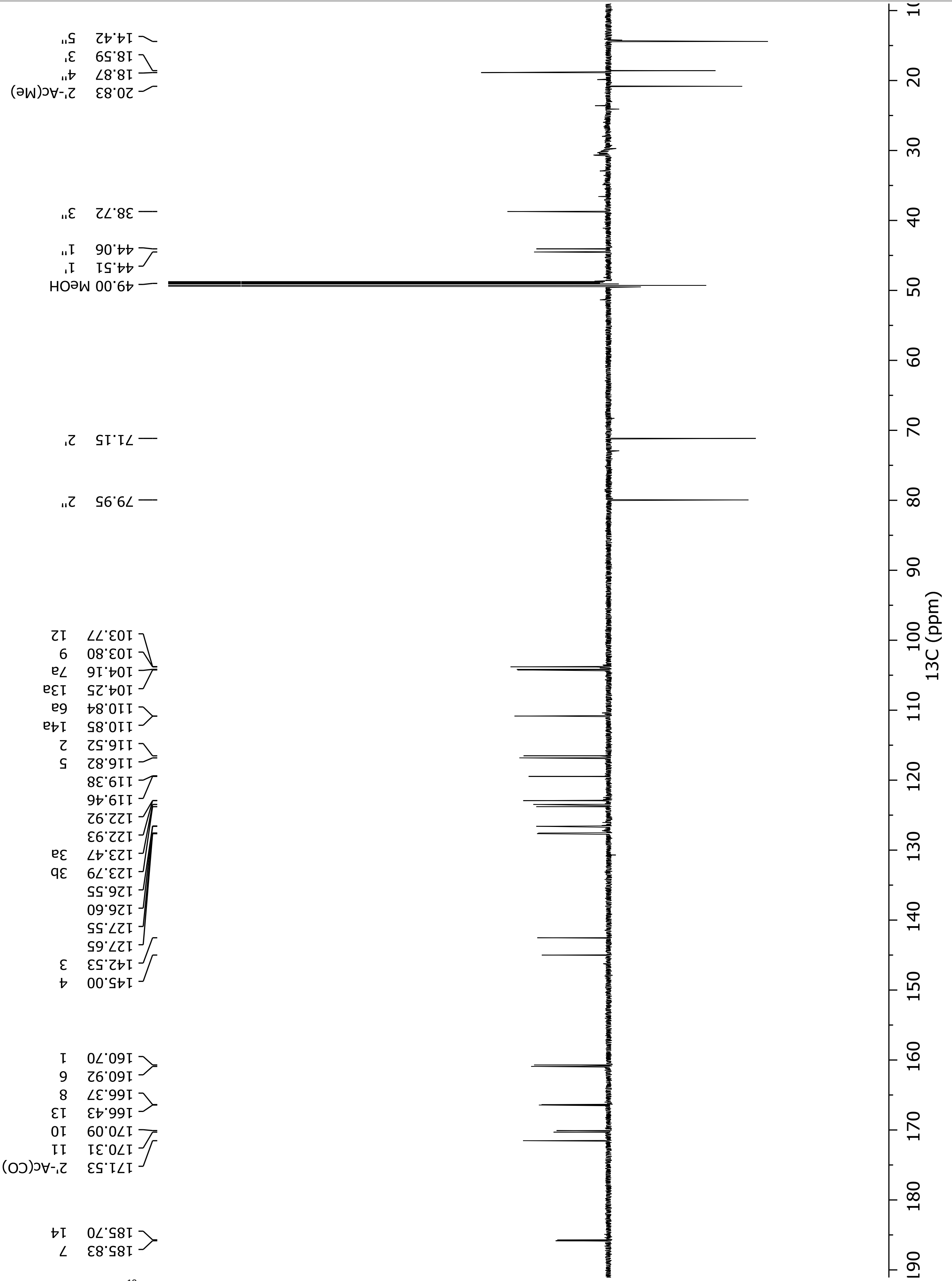

Figure S7. 200MHz ^{13}C NMR spectrum (*J*-modulated echo) of gymnochrome G.

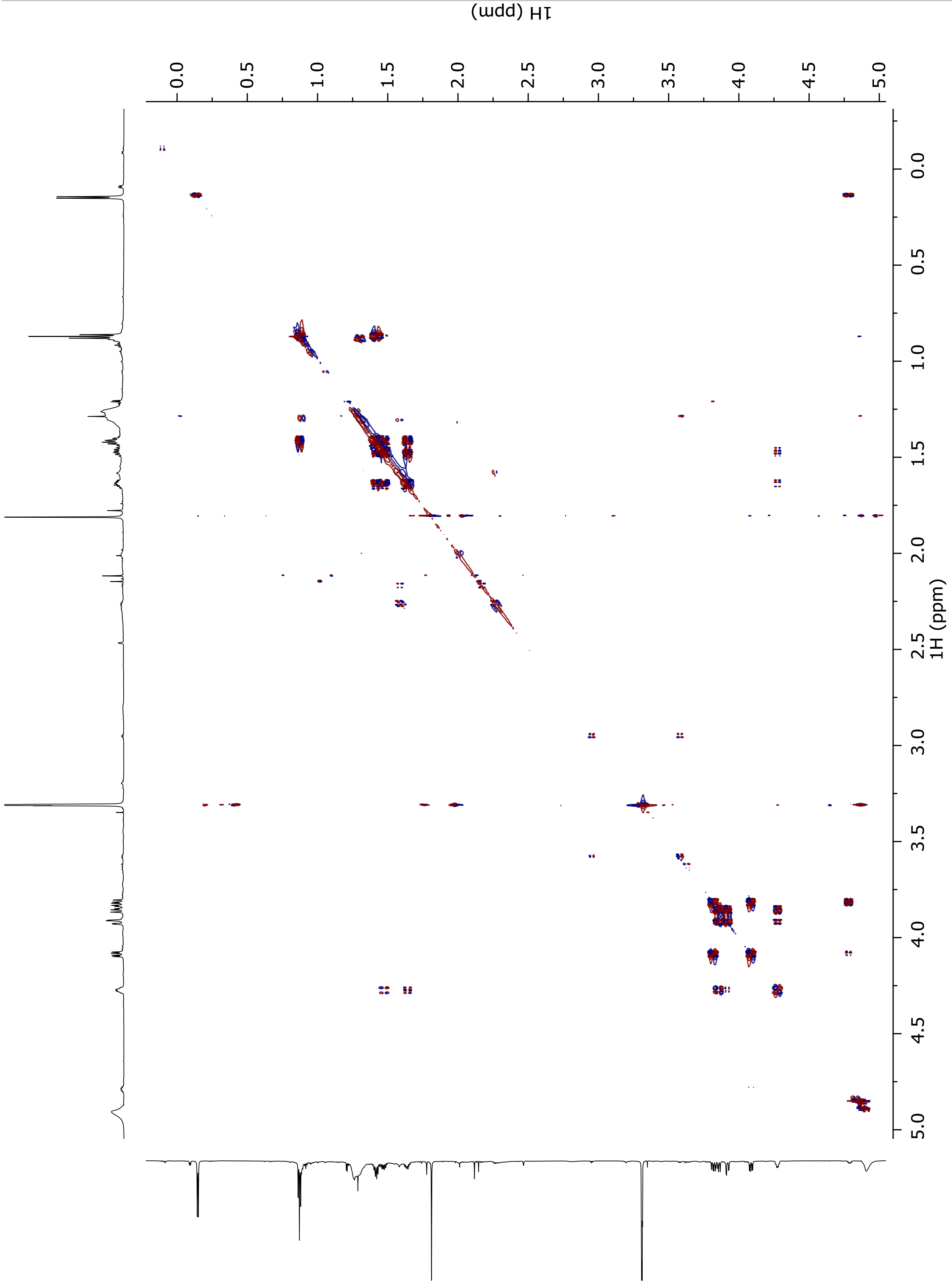

Figure S8. 800MHz DQF-COSY of gymnochrome G.

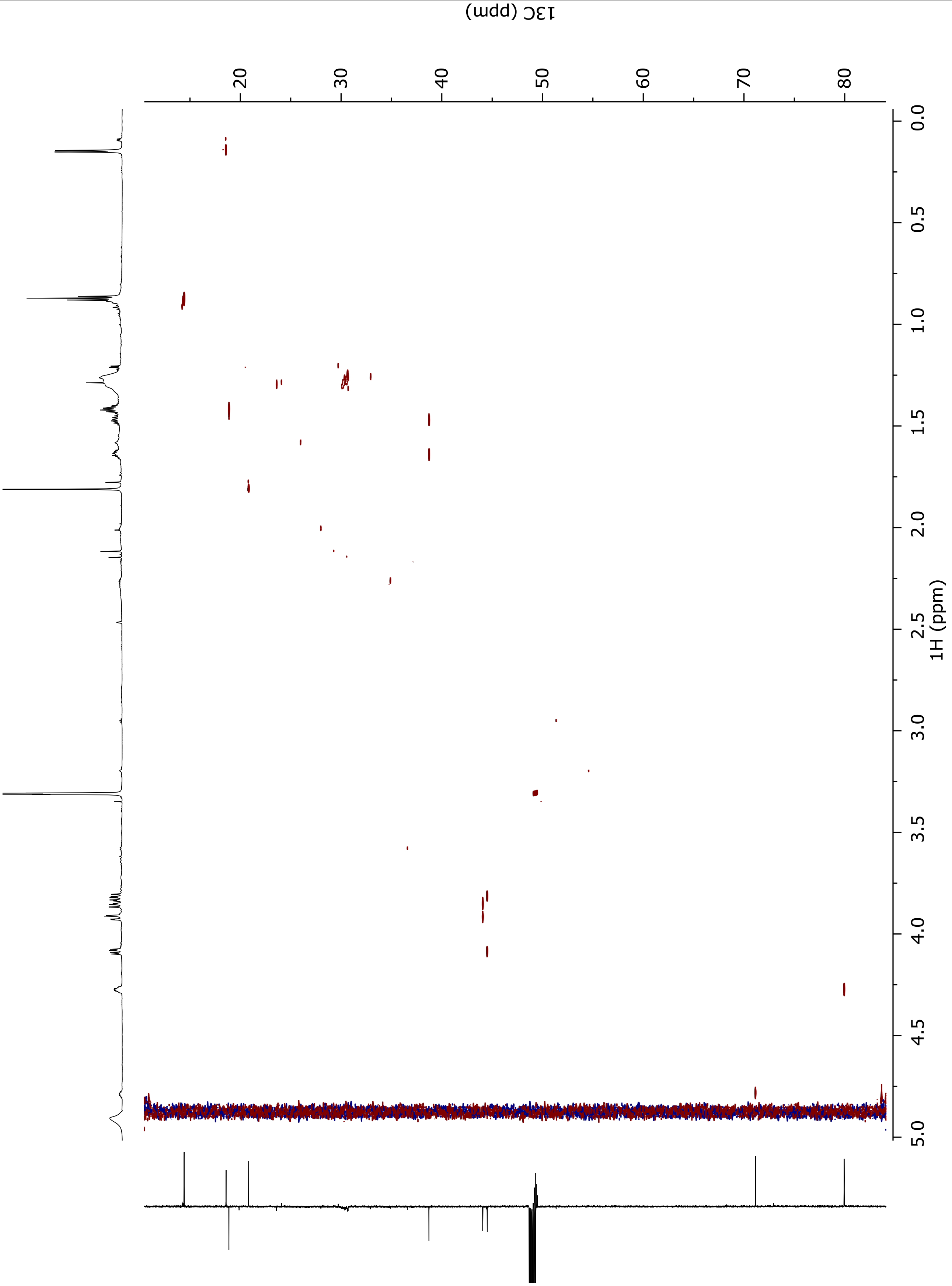

Figure S9. 900MHz HSQC of gymnochrome G.

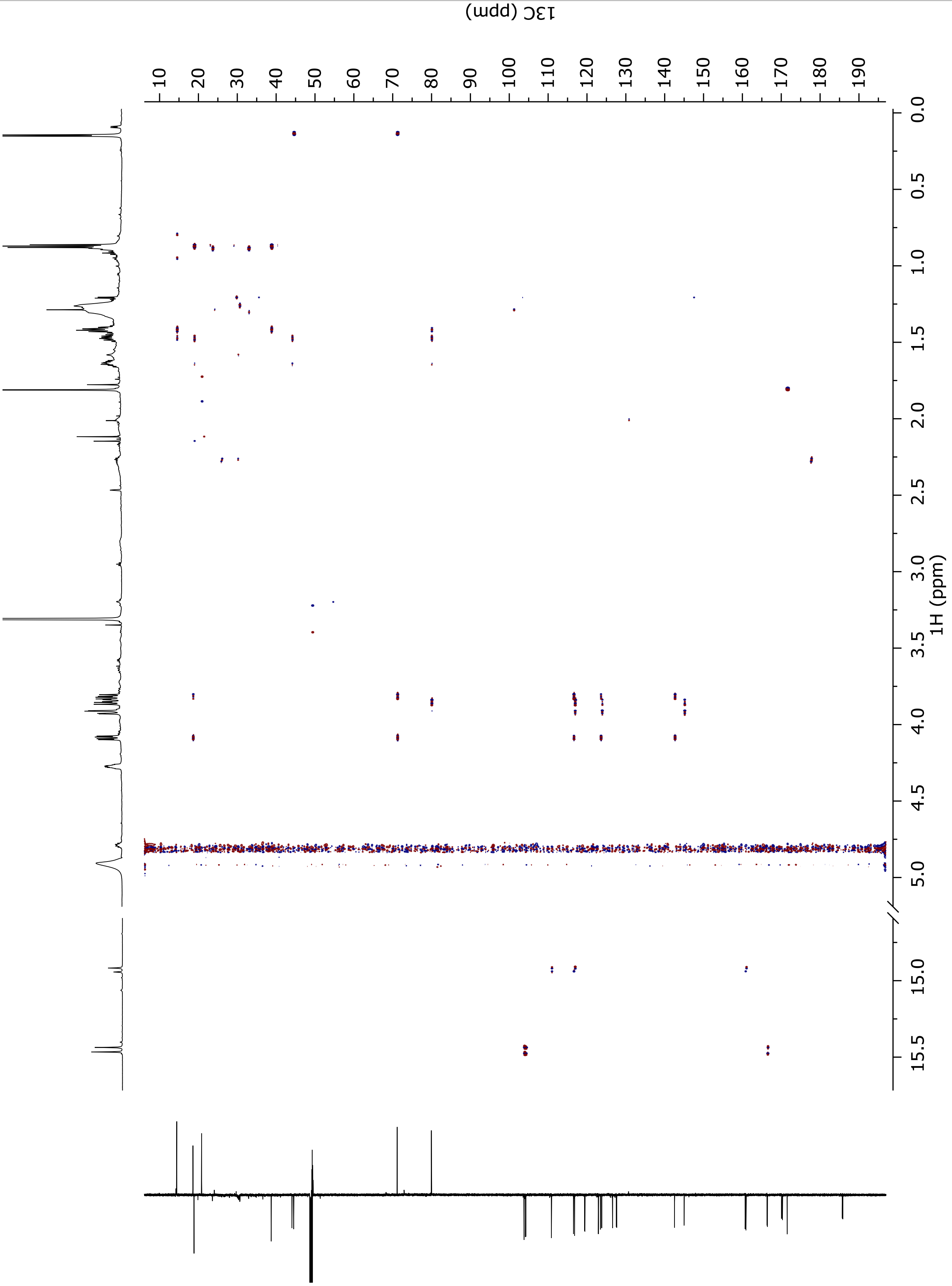

Figure S10. 800MHz HMBC of gymnochrome G.

References

[1] A. Meissner, O. W. Sorensen, *Magn Reson Chem* **2001**, *39*, 49-52.
[2] W. Kozminski, D. Nanz, *J Magn Reson* **2000**, *142*, 294-99.
[3] K. Wolkenstein, J. C. Fuentes-Monteverde, N. Nath, T. Oji, C. Griesinger, *J Nat Prod* **2019**, *82*, 163-67.
[4] N. Grimblat, M. M. Zanardi, A. M. Sarotti, *J Org Chem* **2015**, *80*, 12526-34.
[5] Schrödinger Release 2017-4: Maestro, Schrödinger, LLC, New York, 2019.
[6] Schrödinger Release 2017-4: Macromodel, Schrödinger, LLC, New York, 2019.
[7] T. A. Halgren, *J Comput Chem* **1996**, *17*, 490-519.
[8] G. Chang, W. C. Guida, W. C. Still, *J Am Chem Soc* **1989**, *111*, 4379-86.
[9] a) S. H. Vosko, L. Wilk, M. Nusair, *Can J Phys* **1980**, *58*, 1200-11; b) C. T. Lee, W. T. Yang, R. G. Parr, *Phys Rev B* **1988**, *37*, 785-89; c) A. D. Becke, *J Chem Phys* **1993**, *98*, 5648-52; d) P. J. Stephens, F. J. Devlin, C. F. Chabalowski, M. J. Frisch, *J Phys Chem-Us* **1994**, *98*, 11623-27.
[10] W. J. Hehre, R. Ditchfield, J. A. Pople, *J Chem Phys* **1972**, *56*, 2257-61.
[11] Gaussian 09, Revision C.01, M. J. Frisch, G. W. Trucks, H. B. Schlegel, G. E. Scuseria, M. A. Robb, J. R. Cheeseman, G. Scalmani, V. Barone, B. Mennucci, G. A. Petersson, H. Nakatsuji, M. Caricato, X. Li, H. P. Hratchian, A. F. Izmaylov, J. Bloino, G. Zheng, J. L. Sonnenberg, M. Hada, M. Ehara, K. Toyota, R. Fukuda, J. Hasegawa, M. Ishida, T. Nakajima, Y. Honda, O. Kitao, H. Nakai, T. Vreven, J. J. A. Montgomery, J. E. Peralta, F. Ogliaro, M. Bearpark, J. J. Heyd, E. Brothers, K. N. Kudin, V. N. Staroverov, T. Keith, R. Kobayashi, J. Normand, K. Raghavachari, A. Rendell, J. C. Burant, S. S. Iyengar, J. Tomasi, M. Cossi, N. Rega, J. M. Millam, M. Klene, J. E. Knox, J. B. Cross, V. Bakken, C. Adamo, J. Jaramillo, R. Gomperts, R. E. Stratmann, O. Yazyev, A. J. Austin, R. Cammi, C. Pomelli, J. W. Ochterski, R. L. Martin, K. Morokuma, V. G. Zakrzewski, G. A. Voth, P. Salvador, J. J. Dannenberg, S. Dapprich, A. D. Daniels, O. Farkas, J. B. Foresman, J. V. Ortiz, J. Cioslowski, D. J. Fox, Gaussian, Inc., Wallingford CT, 2010.
[12] a) C. Adamo, V. Barone, *J Chem Phys* **1998**, *108*, 664-75; b) J. P. Perdew, J. A. Chevary, S. H. Vosko, K. A. Jackson, M. R. Pederson, D. J. Singh, C. Fiolhais, *Phys Rev B* **1992**, *46*, 6671-87.
[13] R. Ditchfield, *Mol Phys* **1974**, *27*, 789-807.
[14] J. Tomasi, B. Mennucci, R. Cammi, *Chem Rev* **2005**, *105*, 2999-3093.
[15] R. Altmann, C. Etzlstorfer, H. Falk, *Monatsh Chem* **1997**, *128*, 785-93.
[16] G. Bifulco, P. Dambruoso, L. Gomez-Paloma, R. Riccio, *Chem Rev* **2007**, *107*, 3744-79.
[17] C. Adamo, V. Barone, *J Chem Phys* **1999**, *110*, 6158-70.
[18] T. Yanai, D. P. Tew, N. C. Handy, *Chem Phys Lett* **2004**, *393*, 51-57.
[19] G. Pescitelli, T. Bruhn, *Chirality* **2016**, *28*, 466-74.
[20] a) T. Bruhn, A. Schaumloffel, Y. Hemberger, G. Bringmann, *Chirality* **2013**, *25*, 243-49; b) SpecDis version 1.71, T. Bruhn, A. Schaumloffel, Y. Hemberger, G. Pecitelli, Berlin, 2017.
[21] E. Debie, E. De Gussem, R. K. Dukor, W. Herrebout, L. A. Nafie, P. Bultinck, *Chemphyschem* **2011**, *12*, 1542-49.
[22] M. Findeisen, T. Brand, S. Berger, *Magn Reson Chem* **2007**, *45*, 175-78.
[23] a) A. Enthart, J. C. Freudenberger, J. Furrer, H. Kessler, B. Luy, *J Magn Reson* **2008**, *192*, 314-22; b) L. Castanar, E. Sistare, A. Virgili, R. T. Williamson, T. Parella, *Magn Reson Chem* **2015**, *53*, 115-19.
[24] G. Bifulco, R. Riccio, G. E. Martin, A. V. Buevich, R. T. Williamson, *Org Lett* **2013**, *15*, 654-57.
[25] F. Jensen, *J Chem Theory Comput* **2014**, *10*, 1074-85.
[26] F. Jensen, *J Chem Theory Comput* **2015**, *11*, 132-38.
[27] F. Weigend, R. Ahlrichs, *Phys Chem Chem Phys* **2005**, *7*, 3297-305.
[28] G. Cornilescu, A. Bax, *J Am Chem Soc* **2000**, *122*, 10143-54.
[29] B. Efron, *Ann Stat* **1979**, *7*, 1-26.
[30] N. Nath, M. Schmidt, R. R. Gil, R. T. Williamson, G. E. Martin, A. Navarro-Vazquez, C. Griesinger, Y. Z. Liu, *J Am Chem Soc* **2016**, *138*, 9548-56.
